# Factors associated with engraftment success of patient-derived xenografts of breast cancer

**DOI:** 10.1186/s13058-024-01794-w

**Published:** 2024-03-21

**Authors:** Jongwon Lee, GunHee Lee, Hye Seon Park, Byung-Kwan Jeong, Gyungyub Gong, Jae Ho Jeong, Hee Jin Lee

**Affiliations:** 1 Department of Pathology, Asan Medical Center, University of Ulsan College of Medicine, 88, Olympic-ro 43-gil, Songpa-gu, Seoul, 05505 South Korea; 2 NeogenTC Corp., Seoul, South Korea; 3 Department of Oncology, Asan Medical Center, University of Ulsan College of Medicine, 88, Olympic-ro 43-gil, Songpa-gu, Seoul, 05505 South Korea

**Keywords:** Breast cancer, Patient-derived xenograft, Engraftment, Deep learning, Artificial intelligence, Morphometrics, Neoadjuvant chemotherapy, Young age, Triple-negative breast cancer

## Abstract

**Background:**

Patient-derived xenograft (PDX) models serve as a valuable tool for the preclinical evaluation of novel therapies. They closely replicate the genetic, phenotypic, and histopathological characteristics of primary breast tumors. Despite their promise, the rate of successful PDX engraftment is various in the literature. This study aimed to identify the key factors associated with successful PDX engraftment of primary breast cancer.

**Methods:**

We integrated clinicopathological data with morphological attributes quantified using a trained artificial intelligence (AI) model to identify the principal factors affecting PDX engraftment.

**Results:**

Multivariate logistic regression analyses demonstrated that several factors, including a high Ki-67 labeling index (Ki-67LI) (*p* < 0.001), younger age at diagnosis (*p* = 0.032), post neoadjuvant chemotherapy (NAC) (*p* = 0.006), higher histologic grade (*p* = 0.039), larger tumor size (*p* = 0.029), and AI-assessed higher intratumoral necrosis (*p* = 0.027) and intratumoral invasive carcinoma (*p* = 0.040) proportions, were significant factors for successful PDX engraftment (area under the curve [AUC] 0.905). In the NAC group, a higher Ki-67LI (*p* < 0.001), lower Miller-Payne grade (*p* < 0.001), and reduced proportion of intratumoral normal breast glands as assessed by AI (*p* = 0.06) collectively provided excellent prediction accuracy for successful PDX engraftment (AUC 0.89).

**Conclusions:**

We found that high Ki-67LI, younger age, post-NAC status, higher histologic grade, larger tumor size, and specific morphological attributes were significant factors for predicting successful PDX engraftment of primary breast cancer.

**Supplementary Information:**

The online version contains supplementary material available at 10.1186/s13058-024-01794-w.

## Introduction

Breast cancer is one of the most commonly diagnosed cancers in women worldwide and it continues to be a significant cause of morbidity and mortality. Despite the numerous advancements in cancer treatment, the heterogeneity and complexity of breast tumors have presented significant obstacles in identifying effective therapies for individual patients. Patient-derived xenograft (PDX) models offer a promising solution to this problem by enabling the testing of novel therapies in preclinical models that more accurately reflect the genetic, phenotypic, and histopathological features of the original tumors. However, establishing PDX models remains a challenging and resource-intensive process. Many factors impact the success of PDX models, including the quality of the tumor sample, the choice of engraftment site, the use of immune-deficient mice, and the timing and method of engraftment [1]. In particular, the low engraftment rate of PDX models has been a major obstacle to their widespread use in preclinical studies [2].

Among all types of tumors, breast cancers have been shown to be particularly challenging when it comes to establishing PDX engraftment. Breast cancer has historically exhibited relatively low but diverse engraftment success rates, ranging from 8 to 77% [3, 4]. Factors associated with successful breast cancer PDX engraftment include applying hormonal supplementation (e.g., estrogen pellets) and using tumor samples with: a higher histologic tumor grade, from specific tumor subtypes (e.g., triple-negative breast cancer [TNBC]), or from metastatic tumors [3, 5–7]. Additionally, selecting appropriate host strains for the specific xenograft type can contribute to improved engraftment outcomes [1].

Artificial intelligence (AI) is emerging as a powerful tool in breast cancer research. Its ability to extract morphometric features from breast cancer histopathology is increasingly being recognized as having great potential [8–11]. Some studies have demonstrated the efficacy of AI in accurately classifying various types of breast cancer, including the automatic detection of invasive ductal carcinoma (IDC) [8, 11].

In this study, we analyzed clinicopathologic factors and quantitatively assessed morphometric features extracted by AI to identify features associated with the success of PDX engraftment of primary breast cancer.

## Materials and methods

### Ethics statement

This research was approved by the Asan Medical Center review board (2016-0935 and 2020-0980). All patients signed informed consent forms prior to participation. All animal studies were conducted in compliance with the guidelines established by the Institutional Animal Care and Use Committee (IACUC) at Asan Medical Center and Ulsan University College of Medicine (2015-12-189, 2018-12-059, and 2020-14-211).

### Patient population and case selection

Patients enrolled in this study had histologically confirmed invasive breast cancer with tumors larger than 1 cm that were detected through physical examination or imaging. Those who achieved radiologic complete remission or a significant reduction in tumor mass following neoadjuvant chemotherapy (NAC) were excluded. A total of 380 surgically resected tumor samples were collected from 2016 to 2021 at Asan Medical Center. These samples were obtained from patients who consented to undergo breast-conserving surgery, mastectomy, axillary lymph node dissection, or metastasectomy. Eight cases were subsequently excluded due to premature deaths of the engrafted mice, leaving 372 breast cancer cases for the final evaluation.

### Clinicopathologic data acquisition

Clinicopathological features were gathered from the patients’ medical records, including surgical pathology reports. The histopathological findings were retrospectively reviewed for all 372 surgical specimens. The pT and pN categories were evaluated based on the 8th edition of the American Joint Committee on Cancer cancer staging system [12]. When NAC was conducted, the residual cancer burden (RCB) and Miller-Payne grade were also assessed.

Estrogen receptor (ER) and progesterone receptor (PR) status were determined through immunohistochemical staining of formalin-fixed, paraffin-embedded tumor tissue sections. Positive staining was defined as nuclear staining in at least 1% of the tumor cells, and the hormone receptor-positive (HR +) type was defined as ER and/or PR IHC-positive.

HER2 status was determined using both immunohistochemistry and, in equivocal cases, silver in situ hybridization according to the guidelines from the American Society of Clinical Oncology/College of American Pathologists [13]. HER2 positivity was defined as IHC 2 + or 3 + , or a SISH amplification ratio of HER2 gene signals to chromosome 17 signals greater than 2.0.

The percentage of tumor cells showing any degree of nuclear staining for Ki-67LI was recorded for each tumor. These percentages were recorded in increments of 10% as follows: 0 for 0–10%, 10 for 10–20%, 20 for 20–30%, 30 for 30–40%, 40 for 40–50%, 50 for 50–60%, 60 for 60–70%, 70 for 70–80%, 80 for 80–90%, and 90 for 90–100%.

TNBC was defined as a subtype of breast cancer in which the tumor cells did not express ER or PR and also did not exhibit overexpression or amplification of HER2.

Histological TIL levels were estimated for all cases using the methods previously published by a TILs working group [14]. TILs were determined by calculating the percentage of the area occupied by mononuclear inflammatory cells within the stromal area of the invasive carcinoma. They were graded with 10% increments as for Ki-67LI.

### In vivo tumor implantation and histopathological analysis

All experiments were performed in accordance with the approved protocol and relevant guidelines and regulations. Detailed procedures are provided in the Supplement Methods section (see Additional file 1).

### AI-assisted morphometric analysis

We developed an AI model for morphometric analysis to extract features for the prediction of PDX engraftment. The model was trained on WSIs of H&E stained surgically resected tissues from 64 breast cancer patients, scanned at 400 × magnification. The dataset was randomly partitioned into a training set (65%) and a testing set (35%). The ResNet50 architecture, pre-trained on the ImageNet dataset, was implemented. Image augmentation techniques such as color normalization, random rotation, and color jittering were applied solely to the training set. The model was trained using a batch size of 256 with a learning rate of 0.0003 and involved fifteen epochs using the Adam optimization algorithm. Fifteen epochs were performed using the Adam optimization algorithm.

**Fig. 1:**
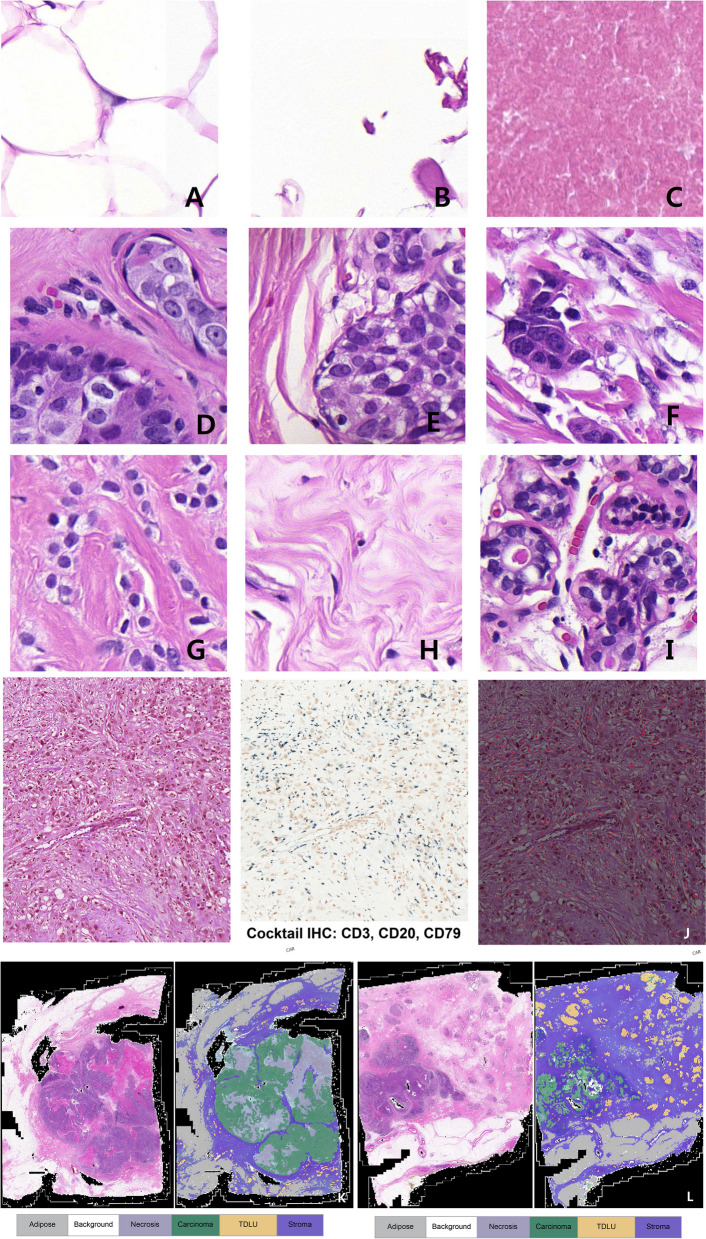

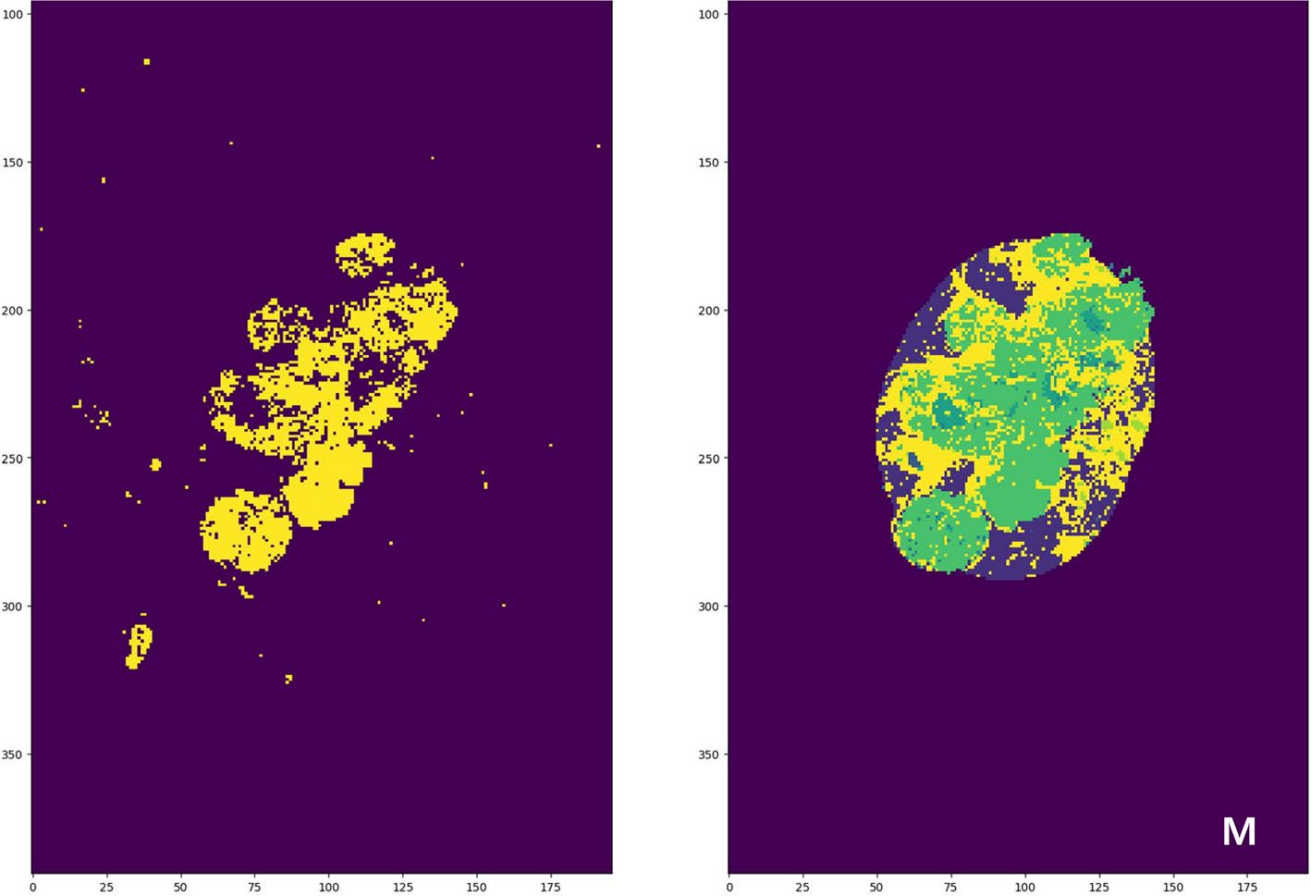
Artificial intelligence-assesed classification of patches. **A**. Adipose; **B**. Background; **C**. Necrosis; **D**. Ductal carcinoma in situ, classified as carcinoma; **E**. Lobular carcinoma in situ, classified as carcinoma; **F**. Invasive ductal carcinoma, classified as carcinoma; **G**. Invasive lobular carcinoma, classified as carcinoma; **H**. Stroma; **I.** Terminal ductal lobular unit; **J.** Tumor-infiltrating lymphocytes trained with a segmentation model. Left, hematoxylin and eosin (H&E) stained slide, X100, original magnification. Middle, Cocktail immunohistochemistry (IHC) for identification of lymphocytes, X100, CD3, CD20, and CD79 cocktail IHC, original magnification. Right, red annotation indicating the area of cocktail IHC-stained lymphocytes in the H&E slides (annotated digitally processed image, original magnification, X100). **K-L**. Representative H&E from successful (**K**) and failed (**L**) PDX graft cases and their corresponding AI-model applied images. **K**. Abundant intratumoral necrosis and carcinoma proportions in the H&E are also highlighted in sky-blue and green in the AI-model applied image, respectively (right); **L.** Abundant intratumoral TILs, stroma, and TDLU identified in the H&E slide (left) are also highlighted in the AI-categorized image (right). **M**. algorithm applied to the carcinoma (left side) generated a tumor boundary (right side, yellow) encircling the carcinoma component (right side, green)

The model processed WSIs into 112 × 112 pixel non-overlapping patches based on morphological similarity. These patches were classified by a consensus meeting between two board-certified pathologists (J.L. and H.L.) into various tissue types, including adipose tissue (Fig. 1A), background (Fig. 1B), and necrosis (Fig. 1C).

Furthermore, we employed a unified classification, categorizing both carcinoma in situ and invasive carcinoma under the umbrella patch label 'carcinoma' (Fig. 1D–G). In terms of other normal structures of the breast parenchyma, patches of stroma (Fig. 1H) and terminal ductal lobular units (TDLUs) (Fig. 1l) were also labeled.

To evaluate TILs, specialized segmentation training was applied to 15 representative breast cancer WSIs. A representative H&E slide was scanned (Fig. 1J, left side), de-stained, and re-stained with a cocktail of immune cell markers (Fig. 1J, middle): CD3 (1:50, Novocastra Laboratories, Leica Biosystems, Nussloch, Germany), CD20 (1:500, Novocastra Laboratories), and CD79 (1:200, Dako, Agilent Technologies, Santa Clara, CA, USA), using a Ventana ES automated immunohistochemistry (IHC) stainer according to the manufacturer’s protocols (Ventana ES automated IHC stainer, Tucson, AZ, USA). Nuclear immunolabeling in black denoted lymphocytes, and they were spatially matched with the H&Es for TIL annotation. These slides were utilized for training of the segmentation model (Fig. 1J, right side) which utilized the ResNet-based DeeplabV3 + . The model was trained for 50 epochs with a learning rate of 0.001 and a batch size of 16. The segmentation model utilized a combination of the ResNet50 architecture for feature extraction and the DeepLabV3 Plus architecture for semantic segmentation. The learning rate was set to 0.001, the batch size was 16, and the model was trained for 50 epochs.

Upon completing the training phase, our AI model was applied to WSIs obtained from 329 out of the 353 surgically resected primary breast cancers that were used for PDX engraftment. The slides were spatially reconstructed for patch interpretation results and color-coded accordingly (Fig. 1K, L). They were then compared to the original H&E stained tumor slides by two pathologists (H.L. and J.L.) for evaluation of the AI-predicted features. The AI model exhibited an F1 score of 0.846 when compared to human pathologists; however, nine slides were excluded from the final analysis because the model had difficulty accurately interpreting carcinoma components; specifically, low histologic grade and sparse cellularity carcinomas were mistakenly identified as TDLU (Additional file 2: Fig. 1A, B) or as stroma (Additional file 2: Fig. 1C).

Finally, 320 slides were selected as candidates for further statistical evaluation. The AI model identified patches within the boundaries of the largest tumor, delineating its edges (Fig. 1M). The proportions of these patches relative to the total number of patches within the tumor perimeter were calculated for the different tissue types, including adipose tissue (adipose tissue intratumoral proportion, AP), necrotic tissue (necrotic tissue intratumoral proportion, NP), terminal ductal lobular units (TDLU intratumoral proportion, TDLUP), stromal tissue (intratumoral proportion of stromal tissue, SP), and invasive carcinoma (invasive carcinoma intratumoral proportion, ICP). ICP was determined by taking the initial estimate of the intratumoral proportions of patches labeled as carcinoma and multiplying by a true invasive carcinoma fraction, which pathologists assessed after a consensus meeting and reviewing all 320 H&E slides.

Tumor-infiltrating lymphocytes intratumoral proportion (TILP) was separately calculated using the aforementioned segmentation model applied within the tumor boundary to determine the area of TILs. The areas identified as lymphocytes were divided by the total area of intratumoral patches. To facilitate statistical analysis and interpretation, these proportions were multiplied by a factor of 10,000 due to the large denominators involved.

### Statistical analysis

Statistical analyses were performed to assess the clinicopathological data regarding the success of PDX engraftment. Independent sample t-tests and Chi-square tests were used to conduct an exploratory analysis of the data. Following this analysis, logistic regression analyses were used to evaluate the clinicopathologic factors that were associated with PDX engraftment. Initially, all potential predictors were included in the univariate logistic regression analyses. A multivariate logistic regression model was subsequently constructed using a backward stepwise elimination process with the goal of optimizing the Akaike Information Criterion (AIC). All variables with a *p*-value less than 0.2 in the univariate analysis were considered for inclusion in the multivariate model. The final multivariate model retained only the variables that contributed to a lower AIC value, ensuring a more parsimonious yet explanatory model. To assess the discriminative ability of the logistic regression models for predicting PDX engraftment, we computed ROC curves and their corresponding area under the curve (AUC) for each model.

Also, recursive partitioning and regression tree classification, as described by Mantzaris et al. [15] were established using the R software package “RPART”. To simplify the model and prevent overfitting, a pruning process was conducted on the initial decision tree. The complexity parameter (CP) was selected based on the printcp output, which evaluated the cross-validated error for different CP values. For our tree analyses, a CP value of 0.03 was chosen for pruning, balancing the model complexity, and the predictive accuracy. In the decision tree models, the variable importance was quantified based on the degree of information gain or impurity reduction each variable contributed to the splits at various nodes, which did not necessarily correspond to the most important variables at each node as displayed in the plotted decision trees.

To evaluate the reliability and stability of the logistic regression models and the RPART tree, bootstrap analyses were conducted. A total of 1000 bootstrap replicates were generated. This resampling procedure was used to estimate the distribution of the AUC. Bias-corrected accelerated (BCa) bootstrap methods were employed to calculate confidence intervals for the AUC. All statistical analyses were conducted using R software version 4.2.1.

### Code availability

The underlying code for this study and training,validation datasets is not publicly available but may be made available to qualified researchers on reasonable request from the corresponding author.

## Results

### Factors affecting the engraftment success rates of primary breast cancer

The clinicopathological characteristics of the primary breast cancer patient population, including both chemo-naive and NAC groups, in relation to the PDX engraftment are summarized in Table 1**,** and the detailed PDX engraftment success rates across multi-passages are described in Additional file 1 and Additional file 2: Fig. 2. In the cohort of 353 primary breast cancer patients, the mean age in the engraftment success group was 45.8 ± 11.0 years, significantly younger than the 50.9 ± 12.2 years observed in the engraftment failure group (*p* = 0.003). A higher prevalence of TNBC was observed in the success group, accounting for 88.1% (52/59) compared to 32.3% (95/294) in the failure group (*p* < 0.001). Ki-67LI (%) was significantly elevated in the success group, with a mean value of 73.2 ± 14.9, compared to 39.0 ± 29.1 in the failure group (*p* < 0.001). In terms of NAC treatment, 79.7% (47/59) of the success group was from the NAC group, which was significantly higher than the 37.1% (109/294) in the failure group (*p* < 0.001). Tumor size was also significantly different, with the success group averaging 4.1 ± 2.6 cm and the failure group averaging 3.3 ± 2.2 cm (*p* = 0.019). The histologic grade was also notably associated with PDX engraftment success, with 91.5% (54/59) of successful cases being histologic grade 3 compared to 48.0% (141/294) in the failure group (*p* < 0.001). Other variables, including diagnosis, LVI, number of positive LNs, TIL%, and AJCC stages showed no significant differences between the success and failure groups.

**Table 1:** Characteristics of the primary breast cancer patient population based on the success of PDX engraftment

Variable	All (n = 353)			NAC group (n = 156)		
	Failure (n = 294)	Success (n = 59)	*p*-value*	Failure (n = 109)	Success (n = 47)	*p*-value*
Age	50.9 ± 12.2	45.8 ± 11.0	**0.003**	50.4 ± 10.7	45.2 ± 11.0	**0.007**
Subtype			**< 0.001**			**< 0.001**
HER2 +	20 (6.8%)	1 (1.7%)		8 (7.3%)	1 (2.1%)	
HR +	147 (50.0%)	5 (8.5%)		48 (44.0%)	4 (8.5%)	
HR + /HER2 +	32 (10.9%)	1 (1.7%)		16 (14.7%)	1 (2.1%)	
TNBC	95 (32.3%)	52 (88.1%)		37 (33.9%)	41 (87.2%)	
Ki-67LI (%)	39.0 ± 29.1	73.2 ± 14.9	**< 0.001**	33.9 ± 32.3	74.0 ± 15.1	**< 0.001**
NAC			**< 0.001**			
Yes	109 (37.1%)	47 (79.7%)		–	–	
No	185 (62.9%)	12 (20.3%)		–	–	
Diagnosis			0.474			0.176
IDC	262 (89.1%)	53 (89.8%)		198(94.7%)	43(91.5%)	
ILC	7 (2.4%)	0 (0.0%)		2(0.96%)	0(0.0%)	
Adenoid cystic carcinoma	1 (0.3%)	1 (1.7%)		0(0.0%)	0(0.0%)	
Invasive apocrine carcinoma	1 (0.3%)	0 (0.0%)		0(0.0%)	0(0.0%)	
Micropapillary carcinoma	8 (2.7%)	2 (3.4%)		5(2.4%)	2(4.3%)	
Metaplastic carcinoma	8 (2.7%)	3 (5.1%)		1(0.5%)	2(4.3%)	
Mucinous carcinoma	7 (2.4%)	0 (0.0%)		3(1.4%)	0(0.0%)	
Size (cm)	3.3 ± 2.2	4.1 ± 2.6	**0.019**	4.3 ± 2.7	4.6 ± 2.7	0.542
LVI			0.376			0.280
Not identified	163 (55.4%)	37 (62.7%)		53 (48.6%)	28 (59.6%)	
Present	131 (44.6%)	22 (37.3%)		56 (51.4%)	19 (40.4%)	
Number of positive LNs	2.7 ± 8.5	3.2 ± 7.3	0.646	5.6 ± 13.1	4.0 ± 8.0	0.336
TIL (%)	11.0 ± 17.9	9.3 ± 13.6	0.43	4.8 ± 10.3	6.6 ± 9.6	0.303
HG			**< 0.001**			**< 0.001**
2	153 (52.0%)	5 (8.5%)		55 (50.5%)	4 (8.5%)	
3	141 (48.0%)	54 (91.5%)		54 (49.5%)	43 (91.5%)	
pT			0.099			0.433
1	74 (25.2%)	10 (16.9%)		16 (14.7%)	5 (10.6%)	
2	170 (57.8%)	36 (61.0%)		57 (52.3%)	29 (61.7%)	
3	47 (16.0%)	10 (16.9%)		33 (30.3%)	10 (21.3%)	
4	3 (1.0%)	3 (5.1%)		3 (2.8%)	3 (6.4%)	
pN			0.396			0.093
0	154 (52.4%)	35 (59.3%)		34 (31.2%)	25 (53.2%)	
1	85 (28.9%)	12 (20.3%)		35 (32.1%)	10 (21.3%)	
2	32 (10.9%)	5 (8.5%)		23 (21.1%)	5 (10.6%)	
3	23 (7.8%)	7 (11.9%)		17 (15.6%)	7 (14.9%)	
M			1.000			1.000
0	291 (99.0%)	58 (98.3%)		106 (97.2%)	46 (97.9%)	
1	3 (1.0%)	1 (1.7%)		3 (2.8%)	1 (2.1%)	
AJCC stage			0.55			0.213
I	55 (18.7%)	7 (11.9%)		12 (11.0%)	4 (8.5%)	
II	162 (55.1%)	35 (59.3%)		43 (39.4%)	27 (57.4%)	
III	75 (25.5%)	16 (27.1%)		51 (46.8%)	15 (31.9%)	
IV	2 (0.7%)	1 (1.7%)		3 (2.8%)	1 (2.1%)	
Miller Payne grade						** < 0.001**
1	-	-		19 (17.4%)	26 (55.3%)	
2	-	-		39 (35.8%)	10 (21.3%)	
3	-	-		46 (42.2%)	11 (23.4%)	
4	-	-		5 (4.6%)	0 (0.0%)	
RCB score	-	-		3.1 ± 1.0	3.2 ± 1.2	0.659
RCB class						0.052
I	-	-		4 (3.7%)	0 (0.0%)	
II	-	-		44 (40.4%)	28 (59.6%)	
III	-	-		61 (56.0%)	19 (40.4%)	

*PDX* Patient-derived xenograft, *NAC* Neoadjuvant chemotherapy, *HER2* HER2 positive breast cancer, *HR +* , hormone receptor-positive breast cancer, *TNBC* Triple-negative breast cancer, *Ki-67LI* Ki-67 labeling index, *IDC* Invasive ductal carcinoma, *ILC* Invavsive lobular carcinoma, *LVI* Lymphovascular invasion, *LN* Lymph node, *TIL* Tumor-infiltrating lymphocytes, *HG* Histologic grade, *pT* Pathological tumor stage, *pN* Pathological nodal stage, *M* Metastasis stage, *AJCC* American Joint Committee on Cancer, *RCB* Residual cancer burden

^*^Bold: significant at *p*-value < 0.05

AI-assessed morphometric features in the cohort of 320 primary breast cancer patients, including both chemo-naive and NAC group patients, were also analyzed, and significant differences were observed between the failure (n = 270) and success groups (n = 50) (Table 2). The success group exhibited a significantly lower average AP (*p* = 0.006). Conversely, the success group had a significantly higher NP compared to the failure group (*p* < 0.001), along with a significantly lower TDLUP (*p* < 0.001). Similarly, the success group had a lower SP than the failure group (*p* = 0.007). Although the ICP was higher in the success group, statstical significance was not reached (*p* = 0.096). There were no significant differences in TILP between the groups.

Both univariate and multivariate logistic regression analyses were conducted, incorporating a range of clinicopathological variables as well as AI-analyzed morphometric features (Table 3). In univariate analysis, several clinicopathologic factors were found to be significantly related to successful PDX engraftment, including younger age (OR 0.96, CI 0.94–0.99, *p* = 0.005), higher Ki-67LI (OR 1.06, CI 1.04–1.08, *p* < 0.001), TNBC subtype (OR 9.78, CI 1.27–75.23, *p* = 0.028), histologic grade 3 (OR 16.62, CI 5.05–54.71, *p* < 0.001), larger invasive tumor size (OR 1.23, CI 1.09–1.38, *p* < 0.001) and more positive metastatic LNs (OR 1.04, CI 1.00–1.08, *p* = 0.031).

**Table 2:** AI-analyzed intratumoral image patch proportions and PDX engraftment success in primary breast cancers

Variable *	All (n = 353)			NAC group (n = 131)		
	Failure (n = 294)	Success (n = 59)	*p*-value**	Failure (n = 93)	Success (n = 38)	*p*-value**
AP	639.9 ± 727.1	409.0 ± 484.0	**0.006**	517.3 ± 622.2	450.9 ± 528.0	0.564
NP	740.7 ± 981.9	1575.0 ± 1264.1	** < 0.001**	901.3 ± 1101.4	1642.8 ± 1358.9	**0.001**
TDLUP	504.4 ± 692.5	219.8 ± 233.3	** < 0.001**	475.2 ± 730.2	201.6 ± 213.9	**0.001**
SP	3377.5 ± 1265.9	2855.3 ± 1094.8	**0.007**	3512.4 ± 1523.4	2855.7 ± 1180.3	**0.019**
TILP	17.8 ± 22.8	15.0 ± 15.9	0.285	10.2 ± 14.0	11.3 ± 13.3	0.678
ICP	4208.0 ± 1450.8	4571.6 ± 1201.5	0.096	3056.0 ± 1470.1	3271.5 ± 1174.1	0.423

*AP* Adipose proportion, *NP* Necrosis proportion, *BP* Background proportion, *TDLUP* Terminal ductal lobular unit proportion, *SP* Stroma proportion, *TILP* Tumor-infiltrating lymphocyte proportion, *ICP* Invasive carcinoma proportion;

^*^Values scaled up by 10,000

^**^Bold: significant at *p*-value < 0.05

**Table 3:** Logistic regression analyses of clinicopathologic factors and AI-analyzed image data impacting PDX engraftment

		All (n = 320)		Univariate ** (Odds ratio, 95% CI, *p*-value***)	Multivariate ** (Odds ratio, 95% CI, *p*-value***)			NAC group (n = 131)		Univariate ** (Odds ratio, 95% CI, *p*-value***)	Multivariate** (Odds ratio, 95% CI, *p*-value***)
Variable*		Failure (n = 270)	Success (n = 50)			Variable*		Failure (n = 93)	Success (n = 38)		
AP	Mean ± SD	639.9 ± 727.1	409.0 ± 484.0	**0.61 (0.47–0.79, *p* = 0.035)**		AP	Mean ± SD	517.3 ± 622.2	450.9 ± 528.0	0.82 (0.74–0.91, *p* = 0.562)	
NP	Mean ± SD	740.7 ± 981.9	1575.0 ± 1264.1	**1.58 (1.39–1.80, *p* < 0.001)**	**1.927 (1.077–3.449, *p* = 0.027)**	NP	Mean ± SD	901.3 ± 1101.4	1642.8 ± 1358.9	**1.60 (1.38–1.85, *p* = 0.003)**	
TDLUP	Mean ± SD	504.4 ± 692.5	219.8 ± 233.3	**0.18 (0.08–0.41, *p* = 0.006)**		TDLUP	Mean ± SD	475.2 ± 730.2	201.6 ± 213.9	**0.22 (0.18–0.27, *p* = 0.034)**	0.181 (0.033–1.00, *p* = 0.06)
SP	Mean ± SD	3377.5 ± 1265.9	2855.3 ± 1094.8	**0.63 (0.46–0.86, *p* = 0.007)**	1.545 (0.844–2.827, *p* = 0.159)	SP	Mean ± SD	3512.4 ± 1523.4	2855.7 ± 1180.3	**0.71 (0.60–0.84, *p* = 0.022)**	
TILP	Mean ± SD	17.8 ± 22.8	15.0 ± 15.9	0.00 (0–7833.4, *p* = 0.401)		TILP	Mean ± SD	10.2 ± 14.0	11.3 ± 13.3	283.00 (29.48–2718.95, *p* = 0.676)	
ICP	Mean ± SD	4208.0 ± 1450.8	4571.6 ± 1201.5	1.19 (0.97–1.45, *p* = 0.097)	**1.820 (1.028–3.223, *p* = 0.040)**	ICP	Mean ± SD	4105.8 ± 1552.8	4505.6 ± 1187.3	1.00 (1.00–1.00, *p* = 0.159)	
Age	Mean ± SD	50.9 ± 12.1	45.6 ± 10.4	**0.96 (0.94–0.99, *p* = 0.005)**	**0.96 (0.92–1.00, *p* = 0.032)**	Age	Mean ± SD	49.6 ± 10.3	45.6 ± 11.0	0.96 (0.93–1.00, *p* = 0.053)	
Ki-67LI (%)	Mean ± SD	41.2 ± 28.7	74.0 ± 14.3	**1.06 (1.04–1.08, *p* < 0.001)**	**1.05 (1.02–1.07, *p* < 0.001)**	Ki-67LI (%)	Mean ± SD	39.2 ± 32.5	75.0 ± 14.3	**1.06 (1.03–1.09, *p* < 0.001)**	**1.07 (1.03–1.10, *p* < 0.001)**
Subtype	HER2 +	20 (7.4%)	1 (2%)			Subtype	HER2 +	8 (8.6%)	1 (2.6%)		
	HR +	130 (48.1%)	4 (8%)	0.62 (0.07–5.79, *p* = 0.671)			HR +	35 (37.6%)	3 (7.9%)	0.69 (0.06–7.48, *p* = 0.757)	
	HR + /HER2 +	30 (11.1%)	1 (2%)	0.67 (0.04–11.29, *p* = 0.779)			HR + /HER2 +	14 (15.1%)	1 (2.6%)	0.57 (0.03–10.43, *p* = 0.706)	
	TNBC	90 (33.3%)	44 (88%)	**9.78 (1.27–75.23, *p* = 0.028)**			TNBC	36 (38.7%)	33 (86.8%)	7.33 (0.87–61.82, *p* = 0.067)	
Diagnosis	IDC	250 (92.6%)	45 (90%)			Diagnosis	IDC	87 (93.5%)	34 (89.5%)		
	Other	20 (7.4%)	5 (10%)	1.39 (0.50–3.89, *p* = 0.532)			Other	6 (6.5%)	4 (10.5%)	1.71 (0.45–6.42, *p* = 0.430)	
HG	2	139 (51.5%)	3 (6%)			HG	2	41 (44.1%)	3 (7.9%)		
	3	131 (48.5%)	47 (94%)	**16.62 (5.05–54.71, *p* < 0.001)**	**4.34 (1.08–17.53, *p* = 0.039)**		3	52 (55.9%)	35 (92.1%)	**9.20 (2.64–32.05, *p* < 0.001)**	
NAC	No	177 (65.6%)	12 (24%)			Miller-Payne grade	Mean ± SD	2.3 ± 0.8	1.6 ± 0.8	**0.34 (0.20–0.57, *p* < 0.001)**	**0.30 (0.16–0.58, *p* < 0.001)**
	Yes	93 (34.4%)	38 (76%)	**6.03 (3.01–12.09, *p* < 0.001)**	**3.27 (1.41–7.60, *p* = 0.006)**	RCB score	Mean ± SD	3.1 ± 1.0	3.4 ± 1.2	1.24 (0.86–1.78, *p* = 0.253)	
Size (cm)	Mean ± SD	3.1 ± 2.0	4.4 ± 2.8	**1.23 (1.09–1.38, *p* < 0.001)**	**1.20 (1.02–1.41, *p* = 0.029)**	Size (cm)	Mean ± SD	4.0 ± 2.6	5.0 ± 2.9	1.13 (0.99–1.30, *p* = 0.064)	
LVI	No	151 (55.9%)	30 (60%)			LVI	No	44 (47.3%)	21 (55.3%)		
	Yes	119 (44.1%)	20 (40%)	0.85 (0.46–1.56, *p* = 0.594)			Yes	49 (52.7%)	17 (44.7%)	0.73 (0.34–1.55, *p* = 0.410)	
Metastatic LNs	Mean ± SD	2.1 ± 5.8	4.5 ± 9.5	**1.04 (1.00–1.08, *p* = 0.031)**		Metastatic LNs	Mean ± SD	4.1 ± 8.9	5.8 ± 10.6	1.02 (0.98–1.06, *p* = 0.350)	
TIL (%)	Mean ± SD	12.0 ± 18.4	9.4 ± 13.8	0.99 (0.97–1.01, *p* = 0.351)		TIL (%)	Mean ± SD	6.1 ± 11.6	6.3 ± 8.5	1.00 (0.97–1.04, *p* = 0.928)	

*AP* Adipose proportion, *NP* Necrosis proportion, *BP* Background proportion, *TDLUP* Terminal ductal lobular unit proportion, *SP* Stroma proportion, *TILP* Tumor-infiltrating lymphocyte proportion, *ICP* Invasive carcinoma proportion, *Ki-67LI* Ki-67 labeling index, *HR +* Hormone receptor-positive breast cancer, *TNBC* Triple-negative breast cancer, *HR + /HER2 +* Hormone receptor and HER2 positive breast cancer, *HER2 +* HER2 positive breast cancer, *IDC* Invasive ductal carcinoma, *HG* Histologic grade, *NAC* Neoadjuvant chemotherapy, *LVI* Lymphovascular invasion, *LN* Lymph node, *TIL* Tumor infiltrating lymphocytes

^*^AP, BP, NP, TDLUP, SP, TILP, ICP values scaled up by 10,000

^**^AP, BP, NP, TDLUP, SP, TILP, ICP: OR calculated with 1,000-unit change, corresponding to 0.1% of intratumoral percentage adjustment

^***^Bold: significant at *p*-value < 0.05

 Also in the univariate logistic regression analyses, several morphological attributes showed statistical significance. A 0.1% increase in NP increased the odds of PDX engraftment by 58% (NP: OR 1.58, CI 1.39–1.80, *p* < 0.001). Conversely, a 0.1% increase in AP was associated with a 39% decrease in the odds of PDX engraftment (OR 0.61, CI 0.47–0.79, *p* = 0.035). A 0.1% increase in TDLUP resulted in an 82% decrease in PDX engraftment odds (OR 0.18, CI 0.08–0.41, *p* = 0.006), and a 0.1% increase in SP led to a 37% reduction in engraftment odds (OR 0.63, CI 0.46–0.86, *p* = 0.007).

**Fig. 2:**
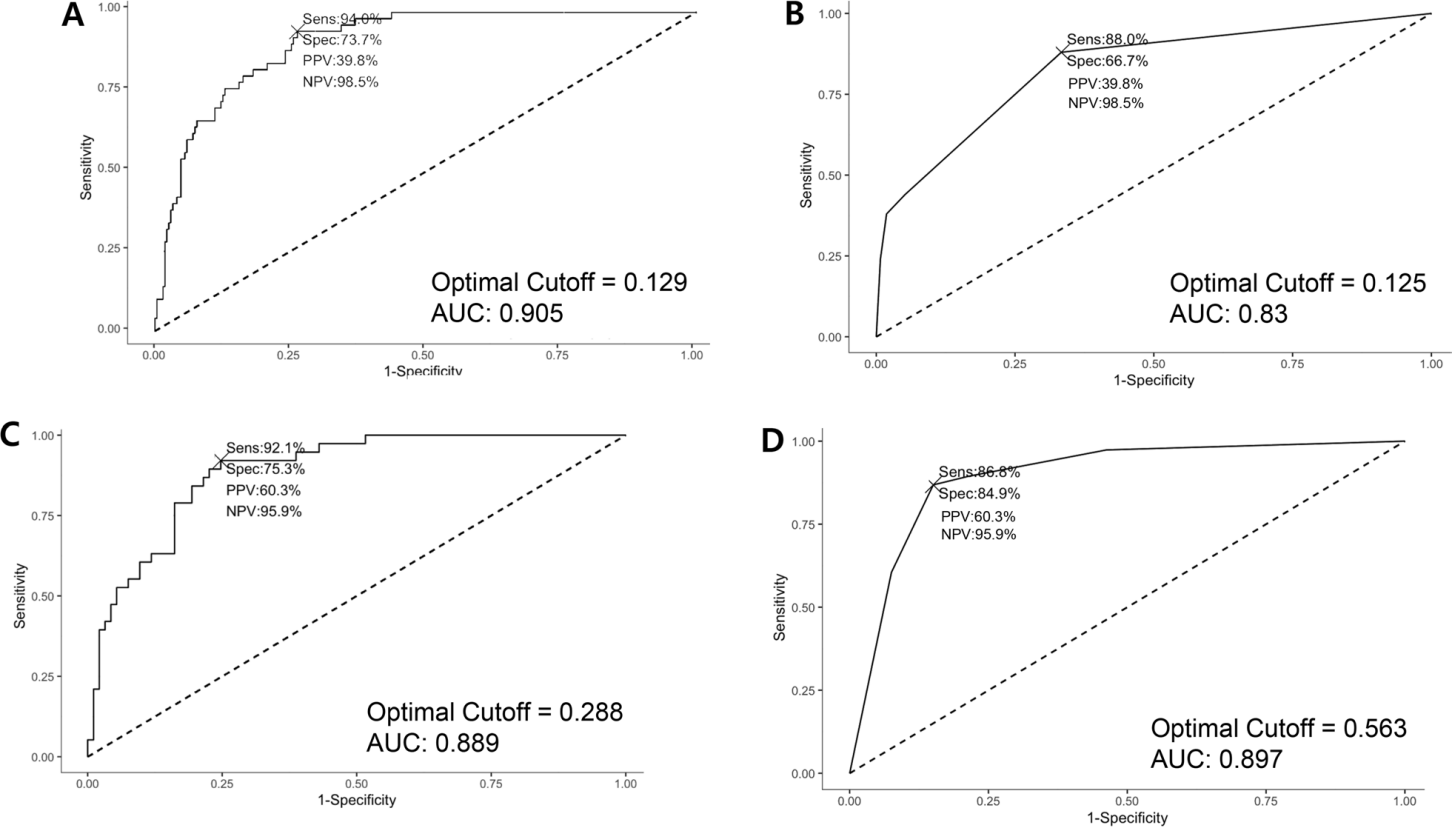
Receiver-operated curves for predictive models of engraftment success. **A**. Multivariate logistic regression of primary breast cancer patients (chemo-naïve and neoadjuvant chemotherapy (NAC)-treated groups), incorporating the selected variables from both AI-evaluated morphometric features and clinicopathological findings. **B**. Pruned decision tree prediction model using clinicopathological and AI-derived morphometric features for primary breast cancer patients (chemo-naïve and NAC-treated groups). **C**. Multivariate logistic regression model for the NAC-treated group. incorporating the selected variables from both AI-evaluated morphometric features and clinicopathological findings. **D**. Pruned decision tree prediction model in the NAC group, utilizing both clinicopathological and AI-derived morphometric features

In multivariate logistic regression analysis of primary breast cancer patients, including the chemo-naïve and NAC groups, variables such as age, Ki-67LI, NAC status, tumor size, histologic grade, NP, ICP, and SP were selected using the stepwise elimination method to achieve the optimal AIC. In the clinicopathologic analysis, significant factors for PDX engraftment included younger age (OR 0.96, CI 0.92–1.00, *p* = 0.032), higher Ki-67LI (OR 1.05, CI 1.02–1.07, *p* < 0.001), NAC status (OR 3.27, CI 1.41–7.60, *p* = 0.006), larger tumor size (OR 1.20, CI 1.02–1.41, *p* = 0.029), and histologic grade 3 (OR 4.34, CI 1.08–17.53, *p* = 0.039). In the analysis of morphometric features, a 0.1% increase in NP increased the odds of success by 92.7% (OR 1.927, CI 1.077–3.449, *p* = 0.027), and a 0.1% increase in ICP increased the odds of success by 82.0% (OR 1.820, CI 1.028–3.223, *p* = 0.040). A bootstrap analysis was conducted to validate the multivariate logistic regression analysis predictive model with the generation of 1000 replicates to assess its reliability. The initial AUC was 0.905, with a bias of 0.0079 and a standard error of 0.0184. The 95% BCa confidence interval for the AUC ranged from 0.8337 to 0.9296. The optimal cutoff point, determined by Youden's J statistic, was 0.129. At this cutoff, the PPV was 0.40 and the NPV was 0.99 (Fig. 2A).

We also generated a decision tree model using the RPART algorithm by incorporating both AI-analyzed morphometric and clinicopathological variables for the primary breast cancer group. The model was pruned at various CPs, and the optimal CP was selected based on the minimized cross-validation error.

**Fig. 3:**
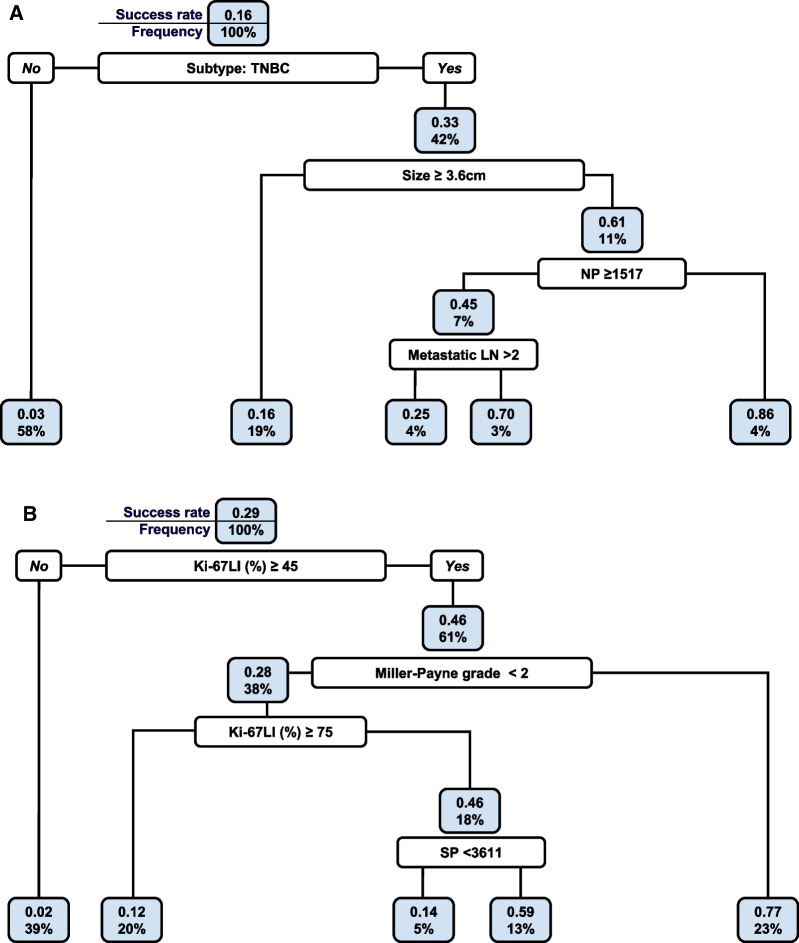
Pruned decision tree analyses. **A** Primary breast cancer, including both chemo-naive and NAC groups (n = 320) and **B** NAC-treated group (n = 131)

In terms of variable importance, cancer subtype was the most significant at 20%, followed by tumor size at 14%, NP at 13%, Ki-67LI at 12%, and patient age at 7%. Additional variables such as SP, metastatic LNs, and histologic grade each contributed 6% to the model (Additional file 3: Table 1 and Fig. 3A**)**.

After bootstrapping with 1000 replicates, the pruned decision tree model yielded an AUC of 0.8304, accompanied by a bias of 0.0694 and a standard error of 0.041. The 95% confidence interval for the AUC, calculated using the BCa method, ranged from 0.6815 to 0.8511. At the optimal cutoff point determined by Youden's J statistic, which was 0.125, the model yielded a PPV of 0.398 and an NPV of 0.985 (Fig. 2B).

### Factors affecting engraftment success rates of NAC-treated primary breast cancer

Separate statistical analyses were carried out for the NAC group with additional inclusion of unique variables, including the Miller-Payne grade, RCB class, and RCB score (Table 1). In the NAC group, samples from 47 of total 156 patients led to successful PDX engraftment (30.1%, 47/156). A significant difference was noted in the mean age between the failure and success groups, with younger age at diagnosis significantly related to PDX engraftment (50.4 ± 10.7 vs. 45.2 ± 11.0 years; *p* = 0.007). The proportion of the TNBC subtype was higher in the success group (87.2%) compared to the failure group (33.9%; *p* < 0.001). Ki-67LI (%) displayed a significant elevation in the success group, registering at 74.0 ± 15.1 compared to 33.9 ± 32.3 in the failure group (*p* < 0.001). The histologic grade also displayed a significant association with PDX engraftment success (*p* < 0.001). Specifically, the successful cases featured a high prevalence of histologic grade 3, accounting for 91.5% (43 out of 47), in contrast to 49.5% (54 out of 109) in the failure group. No significant differences were detected in variables such as diagnosis, LVI, the number of positive LNs, TIL%, tumor size, or AJCC stages between the success and failure groups. The Miller-Payne grade was significantly associated with PDX success (*p* < 0.001). While the RCB score did not show a significant difference, the RCB class showed a statistical trend toward success (*p* = 0.052).

A cohort of 131 NAC-treated primary breast cancers were analyzed using AI-detected morphometric features. These morphometric features were compared between the failure (n = 93) and success (n = 38) groups (Table 2)**.** A notable statistical significance was observed for NP, which was considerably higher in the success group (1642.8 ± 1358.9 vs. 901.3 ± 1101.4; *p* = 0.001). TDLUP showed a significant decrease in the success group compared to the failure group (201.6 ± 213.9 vs. 475.2 ± 730.2; *p* = 0.001). Similarly, SP was significantly lower in the success group (2855.7 ± 1180.3 vs. 3512.4 ± 1523.4; *p* = 0.019).

Univariate and multivariate logistic regression analyses were conducted, incorporating both clinicopathologic factors and AI-analyzed intratumoral image patch data from 131 NAC-treated primary breast cancers (Table 3). In univariate analysis, higher Ki-67LI, histologic grade 3, and lower Miller-Payne grade were found to be significantly associated with PDX engraftment, with odds ratios (ORs) of 1.06 (95% CI 1.03–1.09, *p* < 0.001), 9.20 (95% CI 2.64–32.05, *p* < 0.001), and 0.34 (95% CI 0.20–0.57, *p* < 0.001), respectively. For the morphometric features, higher NP, lesser TDLUP, and lesser SP were significantly associated with PDX engraftment. Specifically, a 0.1% increase in NP was associated with higher odds of successful engraftment (OR 1.60, CI 1.38–1.85, *p* = 0.003), while 0.1% increase in, TDLUP and SP indicated a decreased chance of engraftment (OR 0.22, CI 0.18–0.27, *p* = 0.034 and OR 0.71, CI 0.60–0.84, *p* = 0.022, respectively).

In multivariate logistic regression analysis for the NAC group, the variables TDLUP, Ki-67LI, and Miller-Payne grades were selected as the variables that optimized the AIC. A higher Ki-67LI and lower Miller-Payne grade resulted in successful PDX engraftements with ORs of 1.068 (95% CI 1.034–1.102, *p* < 0.001) and 0.303 (95% CI 0.158–0.577, *p* < 0.001), respectively. A lesser TDLUP was associated with PDX success, with 0.1% increase in TDLUP yielding an OR of 0.998 (95% CI 0.033–1.000, *p* = 0.062), showinga statistical trend.

To evaluate the robustness of the logistic regression model, a bootstrap analysis was conducted using 1000 samples. The original AUC was 0.889, with an associated 95% BCa confidence interval ranging from 0.8204 to 0.9348. The optimal cutoff value for the model, determined by Youden's J statistic, was 0.2863. At this cutoff, the PPV was 0.6034 and the NPV was 0.9589 (Fig. 2C).

In the decision tree for the NAC group incorporating both AI-derived and clinicopathological factors, the tree was pruned at two different CPs, with the optimal CP being 0.0395 as determined by the lowest cross-validation error. The root node error was evaluated at 0.2901, based on 93 failures and 38 successes among the observations (Fig. 3B).

When assessing variable importance, Ki-67LI emerged as the most influential factor, contributing 22% to the model's predictive power. This was followed by Miller-Payne grade (13%), subtype (12%), SP (11%), and histologic grade (9%). Other variables like NP, size, RCB score, and ICP each contributed less than or equal to 5%, whereas variables like LVI, metastatic LNs, TILP, TIL, and age had minimal impact (Additional file 3: Table 2).

The decision tree model for the NAC group demonstrated an AUC of 0.8967, with a bias of 0.0065 and a standard error of 0.0419. The BCa 95% confidence interval for the AUC ranged between 0.7680 and 0.9524 after bootstrapping with 1000 replicates. The recommended cutoff point based on Youden's J statistic was 0.2863, at which the model yielded a PPV of 0.6034 and an NPV of 0.9589 (Fig. 2D).

### Engraftment success across sequential PDX passages and associated clinicopathological factors: metastatectomy cases (n = 19)

The characteristics of the metastatic breast cancers in relation to the success of PDX engraftment are summarized in Additional file 3: Table 3, and Additional file 1**.** A statistically significant difference was observed in the distribution of cancer subtypes between PDX engraftment success and failure groups (*p* = 0.013). Specifically, all successful engraftments occurred for tumors with the TNBC subtype (4 out of 4, 100%) while none of the HR + cases (0 out of 11, 0%) or HR + /HER2 + cases (0 out of 1, 0%) were successful.

The distribution of histologic grade between the unsuccessful and successful PDX groups, although not reaching statistical significance (*p* = 0.134), demonstrated a higher prevalence of grade 3 tumors in the success group at 75% (3/4) compared to 20% (3/15) in the failure group. Although the anatomical site of metastatectomy did not significantly impact PDX engraftment (*p* = 0.207), the successful group displayed a more diverse distribution of metastasis sites: bone at 25% (1/4), axillary lymph nodes at 50% (2/4), and lung at 25% (1/4).

## Discussion

Previous studies on PDX have focused on clinicopathologic or technical parameters for predicting the success of PDX engraftment. In this study, we found that higher Ki-6LI, younger age at diagnosis, NAC treatment status, larger tumor size, and higher proportions of intratumoral necrosis and invasive carcinoma (as quantified by AI) were significant variables predictive of PDX engraftment success.

In this study, high Ki-67LI, a cellular proliferation marker [16–18], emerged as a crucial factor for PDX engraftment. High histologic grade and TNBC subtype, often associated with elevated Ki-67LI, were consistently linked to increased PDX engraftment rates in prior studies [3, 5–7, 19]. These tumors, particularly prevalent in younger patients, reportedly exhibit aggressive behavior and high cancer stem cell (CSC) levels [20, 21].

Increased PDX engraftment rates in the NAC group were also noted in current study. NAC, involving cytotoxic agents, seemed to select cancer cells with survival traits which would largely include CSCs, resistant to NAC due to their ability to evade reactive oxygen species and their non-proliferative nature, as suspected by Diehn and Phi et al. [20, 22]. Substantiating the allegations, post-NAC breast cancer cells showed genetic markers associated with CSCs and high tumorigenic potential in a study by Creighton et al. [23]. Moreover, NAC was observed to modify the tumor microenvironment, including the vascular architecture, which may enhance tumor growth [24]. However, the impact of NAC on PDX engraftment rates in breast cancer remains a subject of debate in the scientific literature. McAuliffe et al. [19] found a significantly higher PDX engraftment rate in the NAC group at 41.7% (10/24) versus 8.3% (2/24) in the chemo-naïve group (*p* = 0.02). Furthermore, in the same study, within the NAC group, patients with progressive disease exhibited higher engraftment rates (85.7%, 6/7) compared to those with a stable or partial response (29.4%, 5/17). However, Goetz et al. [5] found no significant difference in engraftment success between the NAC group and the chemo-naïve group. Cottu et al. [3] also did not find preoperative treatment to significantly influence engraftment success. Thus, factors affected the success of post-NAC cancer PDX grafts should be further evaluated.

While some studies have indicated that metastatic cancers generally have higher PDX engraftment rates compared to their nonmetastatic counterparts [25, 26]., the success rate of PDX engraftment of breast cancer according to region of harvest remains a subject of ongoing debate. Cottu et al. [3] reported higher engraftment success rates with primary breast tumors compared to metastatic samples, while conversely, Marangoni et al. [7] observed increased engraftment success with samples of metastatic origin. Given the limited number of studies comparing primary to metastatic tumors, further research is essential to clarify the role of tumor origin in PDX engraftment success rates. This will provide more comprehensive insights into the factors predictive of successful PDX modeling.

While our study offers valuable insights into the factors affecting PDX engraftment, several limitations need to be acknowledged. First, we faced challenges in differentiating between IDC and DCIS due to the small patch size utilized in our AI model. Given the critical role of tumor boundaries in assessing invasiveness, and thereby differentiating IDC from DCIS, our research necessitated manual intervention. Our approach acknowledges the inherent challenges of integrating machine learning techniques with histopathology [27]. Moving forward, we are committed to refining our strategies in future studies, which will include the use of larger patches to overcome these limitations. Second, our AI model struggled to distinguish between invasive lobular carcinoma, scattered histologic grade 2 IDCs, stromal components, and TDLU. The histopathological complexity and subtle invasion patterns of invasive lobular carcinoma present challenges even for experienced pathologists [28, 29]. These limitations could be addressed in future iterations of our model through the utilization of a larger dataset that encompasses finer patterns of invasive carcinomas and by integrating more sophisticated machine learning techniques.

Our study has shown that the incorporation of specific clinicopathological variables and morphometric features can effectively predict PDX engraftment. The efficacy of our multivariate logistic regression model (AUC = 0.905) is excellent. Additionally, the statistical analysis of the NAC group, which included TDLUP, Miller-Payne grade, and Ki-67LI in the multivariate logistic regression analysis, also demonstrated an excellent predictive ability with an AUC of 0.89. Compared to the PDX graft studies in the literature, our score was higher than that reported by Echeverria et al. who incorporated two variables, Ki-67LI and metastatic LNs, into a logistic regression model for TNBCs breast PDX survival (AUC 0.70) [18]. In the same context, Zhuo et al. [30] used GPC3 expression and KI67LI to predict hepatocellular carcinoma PDX engraftment and found a good discriminatory power (AUC 0.828), which is similar to the score obtained in the current study. Our study also effectively selected a few variables in a parsimonious manner for predicting PDX engraftment.

In conclusion, our research explored the key factors influencing the successful engraftment of breast cancer PDX using various clinicopathological, morphometrical, and statistical methods. Potent clinicopathological factors, including high Ki-67LI, younger patient age, high histologic grade, larger tumor size, and NAC status, notably enhanced engraftment success. Higher proportions of AI-assessed intratumoral necrosis and invasive carcinoma were also associated with successful PDX engraftment. Our study provides valuable suggestions for future research aimed at improving PDX engraftment success, potentially informing preclinical studies and guiding personalized treatment strategies.

### Supplementary Information

**Additional file 1**: Methods of PDX engraftment and engraftment success rates across several passages in both primary and metastatic breast cancer groups.

**Additional file 2**: ** Figure 1**. Failures of identification by the artificial intelligence model. **A**. Histologic grade 2 invasive carcinoma with low cellularity (left and middle side) is mostly assessed as TDLU (right side, yellow). **B**. Residual IDC (middle, arrows) with LVI is mostly interpreted as TDLU (right side, yellow) or stroma (right side, purple). **C. **Low histologic grade IDC with sparse cellularity (middle) is interpreted mostly as stroma (right side, purple). ** Figure 2**. Sequential grafting and engraftment success rates of PDXs. Upper left: P1, involving 394 mice from 372 patients, with successful engraftment observed in 65 mice from 63 patient samples; Upper right: P2, involving further transfer of 61 tumors from P1 into an additional 206 mice, resulting in successful engraftment in 165 mice; Lower left: P3, involving engraftment of tumors from 36 patients into 318 mice, with successful engraftment confirmed in 253 mice; Lower right: P4, involving transfer of tumors from 4 patients into 109 mice, resulting in successful engraftment in 76 mice.

**Additional file 3**: **Table 1**. Decision tree analysis with AI and clinicopathological factors in primary breast cancer group, including the chemo-naïve and NAC groups (n = 320). **Table 2**. Decision tree analysis with AI and clinicopathological factors in the NAC group (n = 131). **Table 3**. Characteristics of the metastatic breast cancer group affecting the success of PDX engraftment (n = 19).

## Data Availability

The datasets used and/or analyzed during the current study are available from the corresponding author on reasonable request.

## References

[1] Chen C (2021). The essential factors of establishing patient-derived tumor model. J Cancer.

[2] Wang H (2017). Establishment of patient-derived gastric cancer xenografts: a useful tool for preclinical evaluation of targeted therapies involving alterations in HER-2, MET and FGFR2 signaling pathways. BMC Cancer.

[3] Cottu P (2012). Modeling of response to endocrine therapy in a panel of human luminal breast cancer xenografts. Breast Cancer Res Treat.

[4] Richard E (2016). The mammary ducts create a favourable microenvironment for xenografting of luminal and molecular apocrine breast tumours. J Pathol.

[5] Goetz MP, et al. Tumor sequencing and patient-derived xenografts in the neoadjuvant treatment of breast cancer. J Natl Cancer Inst. 2017;109(7). 10.1093/jnci/djw306. 10.1093/jnci/djw306 PMC5408989 28376176

[6] Zhang X (2013). A renewable tissue resource of phenotypically stable, biologically and ethnically diverse, patient-derived human breast cancer xenograft models. Cancer Res.

[7] Marangoni E, Poupon MF (2014). Patient-derived tumour xenografts as models for breast cancer drug development. Curr Opin Oncol.

[8] Araújo T (2017). Classification of breast cancer histology images using convolutional neural networks. PLoS One.

[9] Ehteshami Bejnordi B (2017). Diagnostic assessment of deep learning algorithms for detection of lymph node metastases in women with breast cancer. JAMA.

[10] Spanhol FA, et al. Breast cancer histopathological image classification using convolutional neural networks. In: 2016 International Joint Conference on Neural Networks (IJCNN). 2016.

[11] Cruz-Roa A, et al. Automatic detection of invasive ductal carcinoma in whole slide images with convolutional neural networks. In: Medical Imaging. 2014.

[12] Giuliano AE, Edge SB, Hortobagyi GN (2018). Eighth edition of the AJCC cancer staging manual: breast cancer. Ann Surg Oncol.

[13] Wolff AC (2007). American Society of Clinical Oncology/College of American Pathologists guideline recommendations for human epidermal growth factor receptor 2 testing in breast cancer. Arch Pathol Lab Med.

[14] Salgado R (2015). The evaluation of tumor-infiltrating lymphocytes (TILs) in breast cancer: recommendations by an International TILs Working Group 2014. Ann Oncol.

[15] Mantzaris G (2021). DOP60 simplified rules to identify bio-naïve patients with Crohn’s disease with higher likelihood of clinical remission when initiating vedolizumab versus anti-TNFα therapies: analysis of EVOLVE study data. J Crohn's Colitis.

[16] Sittel C (2000). Ki-67 (MIB1), p53, and Lewis-X (LeuM1) as prognostic factors of recurrence in T1 and T2 laryngeal carcinoma. Laryngoscope.

[17] Viale G (2008). Predictive value of tumor Ki-67 expression in two randomized trials of adjuvant chemoendocrine therapy for node-negative breast cancer. J Natl Cancer Inst.

[18] Echeverria GV (2023). Predictors of success in establishing orthotopic patient-derived xenograft models of triple negative breast cancer. npj Breast Cancer.

[19] McAuliffe PF (2015). Ability to generate patient-derived breast cancer xenografts is enhanced in chemoresistant disease and predicts poor patient outcomes. PLoS One.

[20] Phi LTH (2018). Cancer stem cells (CSCs) in drug resistance and their therapeutic implications in cancer treatment. Stem Cells Int.

[21] Charafe-Jauffret E (2010). Aldehyde dehydrogenase 1-positive cancer stem cells mediate metastasis and poor clinical outcome in inflammatory breast cancer. Clin Cancer Res.

[22] Diehn M (2009). Association of reactive oxygen species levels and radioresistance in cancer stem cells. Nature.

[23] Creighton CJ (2009). Residual breast cancers after conventional therapy display mesenchymal as well as tumor-initiating features. Proc Natl Acad Sci U S A.

[24] Mpekris F (2017). Role of vascular normalization in benefit from metronomic chemotherapy. Proc Natl Acad Sci U S A.

[25] Hernandez MC (2019). Patient-derived xenografts can be reliably generated from patient clinical biopsy specimens. J Gastrointest Surg.

[26] Rosfjord E (2014). Advances in patient-derived tumor xenografts: from target identification to predicting clinical response rates in oncology. Biochem Pharmacol.

[27] Komura D, Ishikawa S (2018). Machine learning methods for histopathological image analysis. Comput Struct Biotechnol J.

[28] Linnebacher M (2010). Cryopreservation of human colorectal carcinomas prior to xenografting. BMC Cancer.

[29] Christgen M (2016). Lobular breast cancer: clinical, molecular and morphological characteristics. Pathol Res Pract.

[30] Zhuo J (2021). Molecular phenotypes reveal heterogeneous engraftments of patient-derived hepatocellular carcinoma xenografts. Chin J Cancer Res.

