# Supplementary material for: Factors associated with engraftment success of patient-derived xenografts of breast cancer
Source: Breast Cancer Res. 2024 Mar 21;26:49. doi: 10.1186/s13058-024-01794-w (PMC10956311; doi:10.1186/s13058-024-01794-w)
Supplement: Supplementary file 1 — Additional file 1: Methods of PDX engraftment and engraftment success rates across several passages in both primary and metastatic breast cancer groups. [file 13058_2024_1794_MOESM1_ESM.docx]

**In vivo tumor implantation and histopathological analysis**

Immediately after resection, the tumor specimens were placed in sterile tissue culture medium (RPM 1640) on ice and immediately transported to the animal facility at the Asan Institute for Life Sciences, Asan Medical Center, Seoul, Korea. The tumors were orthotopically implanted into the 4th mammary fat pads of female NOD/SCID mice (5 to 8 weeks old) (Koatech Inc., Seoul, Korea). Estrogen pellets were inserted into the subcutaneous soft tissue of the cervical area of the mouse if the grafted tumor was of an HR-positive subtype. Passage cessation was conducted if a mouse showed any abnormal pathological responses, such as jaundice, extreme distress, or the development of lymphoproliferative disease.

The tumor size was serially measured, and when the diameter of the implanted tumor reached 1 to 2 cm (~2,000 mm^3^ in volume), the mice were euthanized. Then, the tumors were excised, cut into ~2 x 2 x 2 mm fragments, and passaged to successive generations of mice.

We used the retrieved PDXs in three distinct ways. The first group was directly implanted into the next passage of mice in a fresh state. The second group was sampled, fixed in formalin, and then embedded in paraffin blocks. These samples were subsequently stained with hematoxylin and eosin. The stained samples were reviewed for histomorphology by two pathologists (J.L. and H.L.), who compared them with the original slides of the surgically removed tumors that had been grafted. The third group was used to preserve the graft. The tissue was placed in vials containing a mixture of dimethyl sulfoxide and fetal bovine serum (1:9) and kept at -70°C for 24 hours. The vials were then placed in liquid nitrogen tanks (-197°C) for long-term storage.

In the initial passage (P1), direct transplantation into female NOD/SCID mice was conducted, typically involving 1-2 mice per patient sample in the first passage (P1), multiplying the number of mice per patient sample as the passage progressed until the last (4^th^) passage (P4).

**PDX engraftment of primary breast cancer: multi-passage**

The results of PDX engraftments are shown in **Additional File 2: Fig.2**. The first passage (P1) involving grafting 353 distinct primary breast cancer samples into 372 mice. Each mouse received a unique cancer sample, accounting for 336 out of the 353 cancer cases. In the remaining instances, some cancer cases were engrafted into multiple mice. Fifteen cancer cases were each engrafted into two mice, and engraftment success was achieved in five mice from three cases. Moreover, two specific cancer cases were each grafted into three mice; however, successful grafts were not achieved from any of these.

Among the 372 mice included in this passage, we observed 61 instances of successful engraftment, as confirmed by evaluating H&E-stained slides of the engrafted tumors. This indicated an overall success rate of 16.4%, with successful engraftment of 59 out of 353 cases, resulting in a case success rate of 16.7%.

During the second passage (P2), we engrafted 57 primary breast cancer samples into a total of 193 mice, with the number of mice per case ranging from 1 to 15. Of the 193 mice involved, 158 demonstrated successful engraftment, resulting in an overall success rate of 81.9%. Successful engraftment occurred in 52 out of the 57 cases, resulting in a case success rate of 91.2%.

The third passage (P3) involved grafting 33 distinct primary breast cancer cases into 304 mice. This passage displayed a consistently high success rate, with successful engraftment observed for 239 out of 304 mice, representing an overall success rate of 78.6%. We observed successful engraftment of 29 out of 33 cases, resulting in a case success rate of 87.9%.

In the fourth passage (P4), we conducted larger-scale experiments involving four distinct primary breast cancer cases and a total of 102 mice. We observed successful engraftment of 69 out of 102 mice, resulting in an overall success rate of 67.6%. All four cases of breast cancer in P4 resulted in successful engraftment, maintaining a case success rate of 100%.

**Engraftment success across sequential PDX passages and associated clinicopathological factors: metastasectomy cases (n=19)**

In the context of tissue samples from metastatectomies, the first PDX passage included 19 cases, with the majority being single-mouse grafts (16 out of 19), and included a total of 22 mice. This passage yielded an overall engraftment success rate of 18.2% (4/22 mice) and a case-based success rate of 21.1% (4/19 cases). No statistically significant difference in PDX engraftment rate was observed between breast mastectomy and metastatectomy samples (p=0.859).

The second passage involved 4 metastatectomy cases and 13 mice, achieving an overall success rate of 53.8% (7/13 mice) and a case success rate of 75% (3/4 cases).

The third passage included 3 metastasectomy cases engrafted into a total of 14 mice. This passage resulted in a 100% overall success rate (14/14 mice) and a 100% case-based success rate (3/3 cases). Finally, the fourth passage involved a single metastasectomy case and 7 mice, maintaining a 100% success rate both overall (7/7 mice) and at the case level (1/1), underlining consistent performance in this phase.
