# Supplementary material for: Factors associated with engraftment success of patient-derived xenografts of breast cancer
Source: Breast Cancer Res. 2024 Mar 21;26:49. doi: 10.1186/s13058-024-01794-w (PMC10956311; doi:10.1186/s13058-024-01794-w)
Supplement: Supplementary file 2 — Additional file 2: Figure 1. Failures of identification by the artificial intelligence model. A. Histologic grade 2 invasive carcinoma with low cellularity (left and middle side) is mostly assessed as TDLU (right side, yellow). B. Residual IDC (middle, arrows) with LVI is mostly interpreted as TDLU (right side, yellow) or stroma (right side, purple). C. Low histologic grade IDC with sparse cellularity (middle) is interpreted mostly as stroma (right side, purple). Figure 2. Sequential grafting and engraftment success rates of PDXs. Upper left: P1, involving 394 mice from 372 patients, with successful engraftment observed in 65 mice from 63 patient samples; Upper right: P2, involving further transfer of 61 tumors from P1 into an additional 206 mice, resulting in successful engraftment in 165 mice; Lower left: P3, involving engraftment of tumors from 36 patients into 318 mice, with successful engraftment confirmed in 253 mice; Lower right: P4, involving transfer of tumors from 4 patients into 109 mice, resulting in successful engraftment in 76 mice. [file 13058_2024_1794_MOESM2_ESM.pptx]

## Slide 1
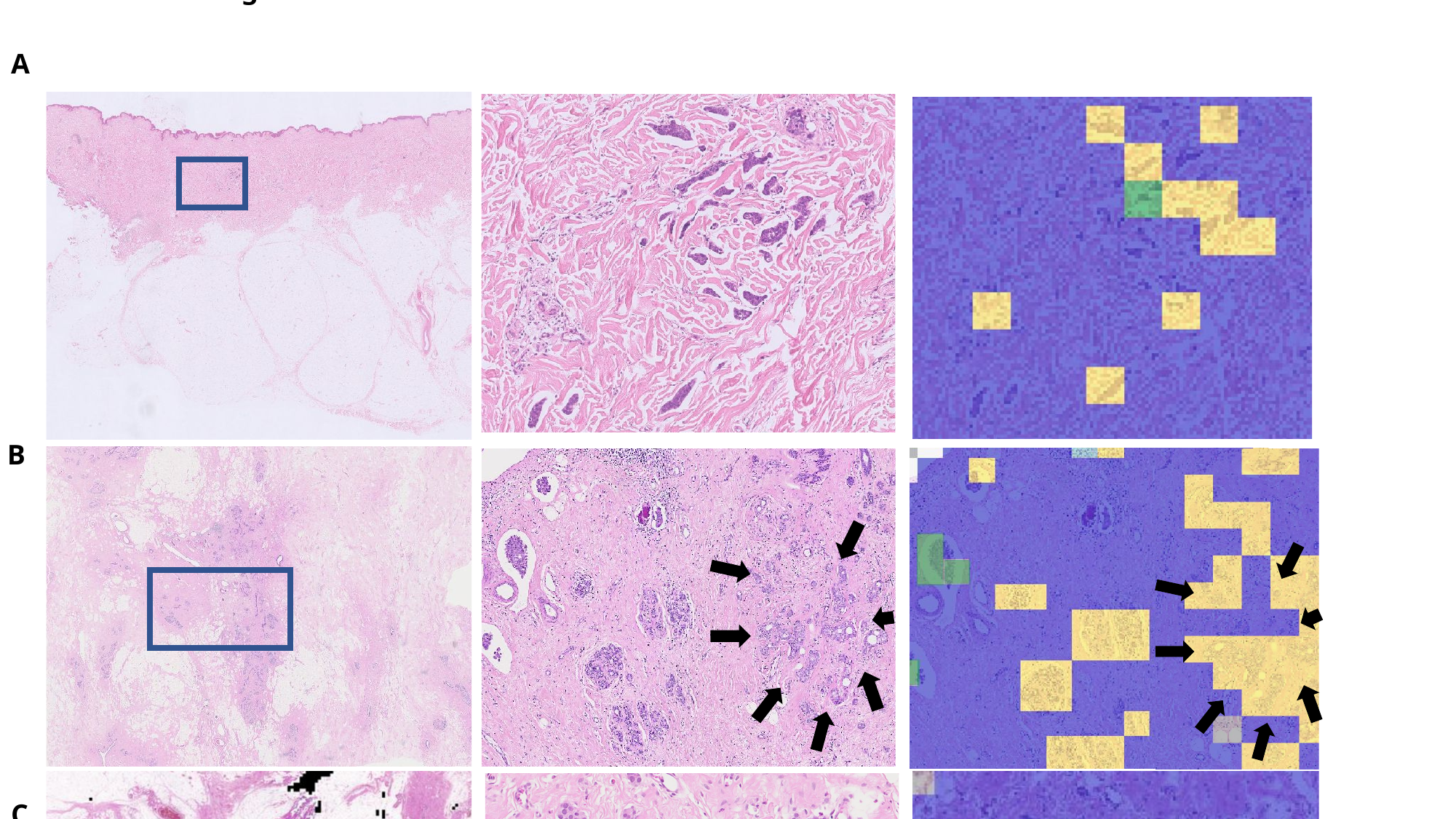

Additional File: Fig.1
A
B
C
| Adipose | Background | Necrosis | Carcinoma | TDLU | Stroma |
| --- | --- | --- | --- | --- | --- |

## Slide 2
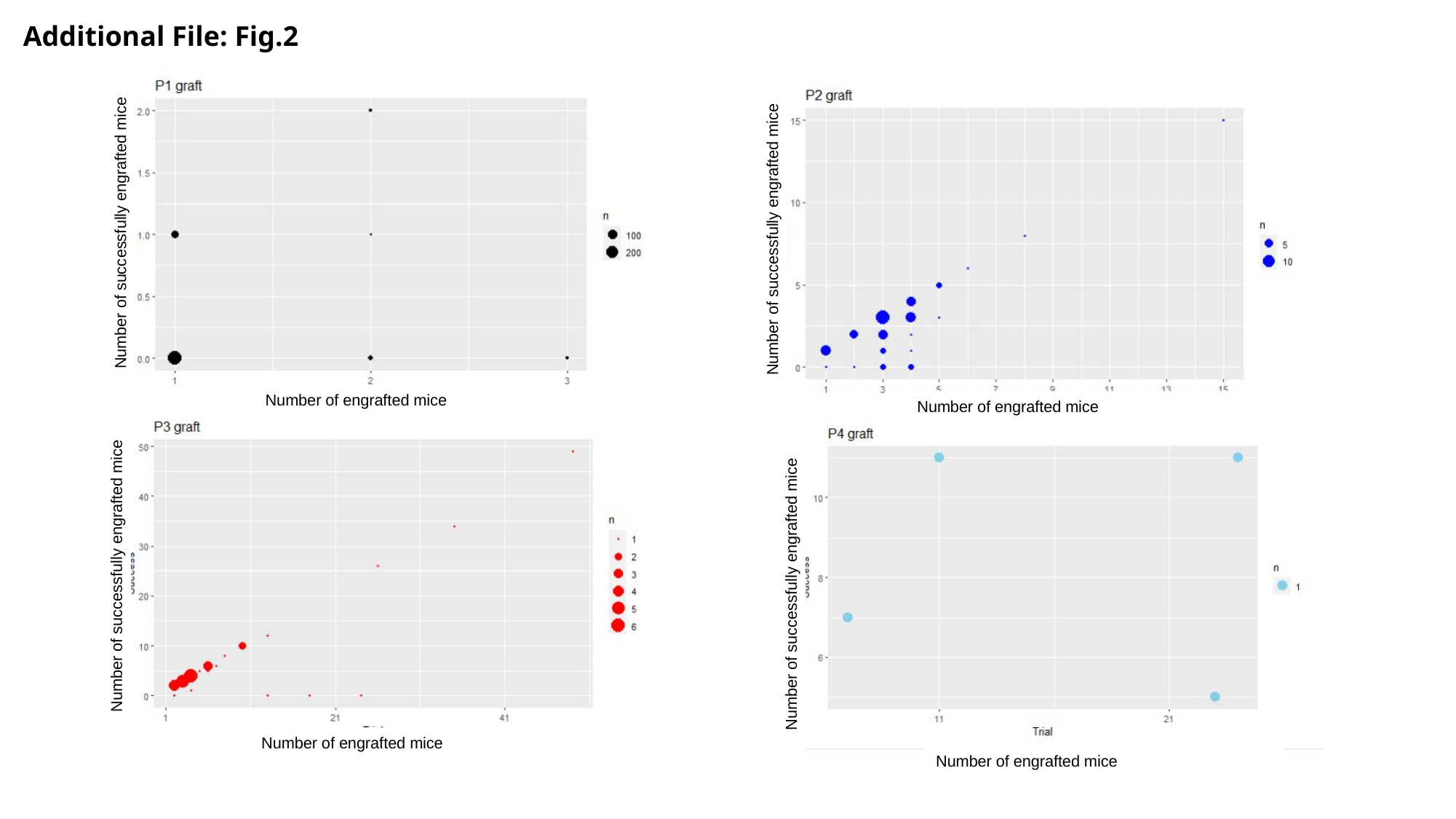

Additional File: Fig.2
Number of successfully engrafted mice
Number of successfully engrafted mice
Number of engrafted mice
Number of engrafted mice
Number of successfully engrafted mice
Number of successfully engrafted mice
Number of engrafted mice
Number of engrafted mice
